# Androgen Receptor Signaling in Prostate Cancer and Therapeutic Strategies

**DOI:** 10.3390/cancers13215417

**Published:** 2021-10-28

**Authors:** Aasems Jacob, Rishi Raj, Derek B. Allison, Zin W. Myint

**Affiliations:** 1 Department of Medicine, Division of Hematology & Oncology, Pikeville Medical Center, Pikeville, KY 41501, USA; aasems.jacob@pikevillehospital.org; 2 Department of Medicine, Division of Endocrinology, Diabetes & Metabolism, Pikeville Medical Center, Pikeville, KY 41501, USA; Rishi.Raj@pikevillehospital.org; 3 Markey Cancer Center, University of Kentucky, Lexington, KY 40536, USA; Derek.Allison@uky.edu; 4 Department of Pathology and Laboratory Medicine, University of Kentucky, Lexington, KY 40536, USA; 5 Department of Urology, University of Kentucky, Lexington, KY 40536, USA; 6 Department of Medicine, Division of Medical Oncology, University of Kentucky, Lexington, KY 40536, USA

**Keywords:** prostate cancer, castrate-resistant growth, metastases, androgen receptor, glucocorticoid receptor, cytokines, transmembrane receptors, cell signaling, pharmacological targeting, neuroendocrine differentiation, lineage plasticity, prostate cancer stem cells

## Abstract

**Simple Summary:**

Early-stage and castration-sensitive prostate cancer (PCa) growth is solely mediated by androgen signaling pathways. AR signaling inhibitors (ARSIs) have significantly improved clinical outcomes among men with PCa. In the metastatic castration-resistant PCa, there is presence of both androgen-dependent and androgen-independent cells driving the tumor growth. Despite the use of ARSIs, disease progression ultimately occurs in all patients with PCa and is due to genetic alterations in ARs, resulting in the outgrowth of androgen-independent cells. The possible mechanisms include development of AR splice variants of which AR-V7 is more common, AR point mutations, and AR overexpression. In addition, restoration of downstream signaling through alternate pathways can also lead to androgen-independent growth of PCa. Therapeutic strategies to overcome these resistance mechanisms and establish predictive biomarkers are still in clinical trials. This review article details the current evidence on clinically relevant driver mechanisms, relevant biomarkers, and treatment modalities to overcome resistance.

**Abstract:**

Understanding of the molecular mechanisms of prostate cancer has led to development of therapeutic strategies targeting androgen receptor (AR). These androgen-receptor signaling inhibitors (ARSI) include androgen synthesis inhibitor-abiraterone and androgen receptor antagonists-enzalutamide, apalutamide, and darolutamide. Although these medications provide significant improvement in survival among men with prostate cancer, drug resistance develops in nearly all patients with time. This could be through androgen-dependent or androgen-independent mechanisms. Even weaker signals and non-canonical steroid ligands can activate AR in the presence of truncated AR-splice variants, AR overexpression, or activating mutations in AR. AR splice variant, AR-V7 is the most studied among these and is not targeted by available ARSIs. Non-androgen receptor dependent resistance mechanisms are mediated by activation of an alternative signaling pathway when AR is inhibited. DNA repair pathway, PI3K/AKT/mTOR pathway, BRAF-MAPK and Wnt signaling pathway and activation by glucocorticoid receptors can restore downstream signaling in prostate cancer by alternative proteins. Multiple clinical trials are underway exploring therapeutic strategies to overcome these resistance mechanisms.

## 1. Introduction

Prostate cancer (PCa) is the second most common malignancy among men [1]. Based on Surveillance, Epidemiology and End Results Program-9 data, the age-adjusted incidence and mortality of PCa for the period 2009 to 2018 showed a downward trend [2]. This drift could be due to a decrease in the utilization of routine prostate specific antigen (PSA) screening and the development of effective therapeutic strategies which prolong PCa survival.

Androgen Receptor (AR) plays a key role in the pathogenesis of PCa. AR is a ligand-activated nuclear transcription factor that belongs to the steroid hormone receptor family. As testosterone (produced by Leydig cells in the testes) or 5-alpha-dihydrotestosterone (DHT) (converted from testosterone in prostate tissue by 5-alpha-reductase type I and II) binds to AR, the receptor dimerizes and translocates into the nucleus to bind to the androgen response element (ARE) [3]. The AR modulates the transcriptional activity of genes involved in escaping apoptosis and inducing cellular proliferation. Thus, AR signaling results in the growth of PCa. A critical level of androgens is needed to activate the required number of ARs on androgen-sensitive PCa cells for cell proliferation, the absence of which by utilizing androgen ablation will result in activation of programmed cell death [4]. Hence, the therapeutic strategy of androgen deprivation therapy (ADT) is rational and beneficial in PCa. Although early-stage PCa is mostly mediated by androgen-dependent cancer cells, the metastatic castrate-resistant stage is heterogenous with the presence of both androgen-dependent and androgen-independent cells. The development of castration resistance is mainly due to the development of genetic alterations in the AR resulting in the outgrowth of androgen-independent cells [5]. In addition to the stimulation of AR by androgen produced from the adrenal gland and testis, intra-tumoral secretion of enzymes involved in the synthesis of testosterone such as cytochrome P450 17-alpha hydroxysteroid dehydrogenase (CYP17) support tumor survival and growth [6]. The understanding of these molecular mechanisms has led to the development of newer drugs that act by inhibiting the enzymes for androgen production or block ARs. The agents include abiraterone which can cause selective and irreversible inhibition of CYP17 and small molecule AR antagonists such as enzalutamide [7]. Although these androgen-receptor signaling inhibitors (ARSI) have been shown to significantly alter the natural history of castration-resistant PCa (CRPC), drug resistance develops in most patients with time. In this article, we will discuss the drugs that target AR and the clinically relevant resistance mechanisms as well as therapeutic strategies to overcome the resistance.

## 2. Therapeutic Strategies Targeted at Androgen Signaling

### 2.1. Androgen Synthesis Inhibitors

Abiraterone acetate is an androgen synthesis inhibitor derived from pregnenolone and is an irreversible inhibitor of 17, 20-lyase and 17-alpha hydroxylase which are products of the CYP17 gene. The drug inhibits the production of androgen in the testes, adrenal glands, and tumor cells [8]. The agent requires steroid supplementation to overcome secondary cortisol insufficiency and prevent overproduction of ACTH and mineralocorticoids [8].

The drug is currently approved by the US Food and Drug Administration (FDA) for use in newly diagnosed metastatic castration-sensitive PCa (CSPC) and metastatic CRPC. Clinical trials that led to FDA approval of individual drugs are shown in Table 1.

### 2.2. Androgen Receptor Antagonists

Androgen receptor antagonists (ARA) block the androgen binding site of AR and inhibit the nuclear translocation of AR and subsequent association of AR with nuclear DNA. This results in attenuation of coactivator mobilization leading to cellular apoptosis and decreased prostate tumor volume. The first-generation ARAs including bicalutamide, nilutamide, and flutamide do not completely block AR activity. Enzalutamide, apalutamide, and darolutamide are the currently utilized second-generation ARAs and have no agonistic activity on AR compared to first-generation ARAs. Enzalutamide and apalutamide have a similar mechanism of action and side effect profile [19]. Darolutamide has lower blood–brain barrier penetration and low binding affinity for gamma-aminobutyric acid type A receptors which resulted in lower incidence of CNS side effects [20]. Clinical trials relevant to each drug are detailed in Table 1.

Enzalutamide is currently approved in de novo mCSPC, non-metastatic CRPC, mCRPC in chemo naïve patients, and mCRPC which progressed after chemotherapy. In addition to overall survival benefit, the medication also improved health-related quality of life and benefited patients above 75 years of age with mCRPC [21,22]. Apalutamide is approved in de novo mCSPC and non-metastatic CRPC. Darolutamide has benefit in improving median metastasis-free survival, overall survival (OS), time to symptomatic skeletal event, and time to chemotherapy in non-metastatic CRPC [20]. Clinical trial in mCSPC is ongoing [23].

Since abiraterone and apalutamide act by different mechanisms, clinical trials are evaluating whether the combination provides superior outcomes. Preliminary reports from abiraterone plus apalutamide combination trial showed improved PSA response while results from abiraterone plus enzalutamide trial showed no improvement in OS and increased side effects [24,25].

In the phase IV Safety Study of Continued Enzalutamide Treatment In Prostate Cancer Patients (PLATO) trial that evaluated 251 patients who were on abiraterone after progression on enzalutamide, the median time to PSA progression (mPSA-P) was 2.8 months, median radiologic progression free survival (rPFS) was 5.7 months, and only 4 patients had PSA decline ≥50% [26,27]. This was redemonstrated in the post hoc analysis of the COU-AA-302 trial where abiraterone after progression on enzalutamide (*n* = 55) gave mPSA-P of only 3.9 months and a PSA decline ≥50% was seen in 44% of the patients [10]. The cohort of patients who had enzalutamide after progression on abiraterone (*n* = 33) had an mPSA-P of 2.8 months and a PSA decline ≥50% in 67% of patients. These studies emphasize the development of AR-mediated cross-resistance which limits the clinical benefit for subsequent use of alternate ARSI.

## 3. Acquired Castration Resistance

Despite the efficacy of ARSI, secondary resistance to these agents develops in nearly all the patients due to molecular changes from selective pressure on AR. The various secondary resistance mechanisms are discussed below with an emphasis on mechanisms that have potential therapeutic implications and are compiled in Table 2.

### 3.1. AR Splice Variants

De novo absence of efficacy with ARSI occurs in approximately 5–10% of mCRPC patients and progression ensues in nearly all the patients who initially respond to these drugs. These failures can be partly attributed to AR variants [55,56,57]. Truncated AR splice variants (AR-V) contain intact activating sites including the N-terminal domain (NTD) and the DNA-binding domain but lack the ligand-binding domain (LBD) where ARAs bind and abiraterone exerts its indirect effect [58]. Based on the type of splice variant, downstream transcriptional activity or AR expression abundance may be affected [59].

Androgen receptor isoform splice variant 7 (AR-V7) is the most common variant detected and is not targeted by available ARSIs [60]. AR-V7 is common in metastatic PCa (75%) and is rare in early-stage disease (<1%), suggesting that the expression adaptively increases in tumors exposed to ARSI. The PROPHECY trial evaluated baseline circulating tumor cell (CTC) AR-V7 among 118 patients prior to initiation of enzalutamide or abiraterone and found that the presence of CTC AR-V7 is associated with shorter PFS and OS, and only 0–11% of patients showed a PSA response compared to 26–28% in AR-V7 negative patients based on the assay used [28,29]. Soft tissue responses were also limited at 0–6% compared to 21–25% in patients without CTC AR-V7. Another larger study of 202 men with mCRPC confirmed the shorter OS in patients with the presence of CTC AR-V7 [28].

Detecting these variants previously required serial biopsies but the use of widely available and validated liquid biopsy has made testing more accessible. Treating physicians should consider testing for AR-V7 in patients who experience disease progression after ARSI. This approach can guide further treatment with an alternative ARSI versus chemotherapy. The presence of CTC AR-V7 is not associated with primary resistance to taxane chemotherapy [31]. Taxanes may be more effective in these patients compared to ARSI while in AR-V7 negative men, both chemotherapy and ARSI (abiraterone or enzalutamide) have comparable efficacy [28]. Combining agents targeted at suppressing or degrading AR-V7 to increase sensitivity to enzalutamide is only in preclinical stages and not implemented in clinical trials [61,62,63].

Although apalutamide and darolutamide target full-length AR with no effect on AR-V7 activity, resistance is seen in AR-V7 expressing enzalutamide- and abiraterone-resistant models. This effect could be mediated by concurrent AKR1C3 enzyme activation in these models which converts weak androgens to the more potent products: testosterone and DHT. AKR1C3 also stabilizes AR-V7 and full-length AR (AR-FL), which results in increased c-MYC expression that in turn activates AR target genes [64,65]. Knockdown of AKR1C3 decreased AR-V7 and c-MYC expression and reversed the cross-resistance to all four agents. Indomethacin is a potent inhibitor of AKR1C3 and is being evaluated as a combination treatment with enzalutamide in mCRPC in a phase I/II trial [66].

TAS3681 is an oral AR antagonist with full length-AR and AR-V7 downregulatory activity and was shown to have antitumor efficacy in enzalutamide-resistant models. The open-label phase I trials among 56 patients refractory to abiraterone (14.2%), enzalutamide (46.4%), or both (39.3%) showed PSA response lasting up to 16.3 months with a manageable safety profile [32]. The expansion phase of the study to assess preliminary efficacy is currently ongoing. It also reduced the expression of c-MYC, an androgen-independent driver of disease progression. The AR-NTD targeting drug EPI-7386 blocks full-length AR and AR-V7 signaling [33]. It is being studied in combination with enzalutamide in men with mCRPC in a phase I trial while EPI-506, which acts by a similar mechanism, did not show significant PSA responses. As a result, the trial was terminated, and the results were attributed to poor pharmacokinetics and considerable pill burden affecting compliance [65].

AR-V567es is another splice variant identified in xenografts after prolonged ADT exposure and increased in enzalutamide-resistant PCa cells [37,38]. CTC ARV-567es was more common than CTC AR-V7 (78% vs. 67%) among 54 patients including 42.6% who received prior ARSI; 54% of patients expressed double positivity. Although taxane chemotherapy improved median PFS in CTC ARV-567es+ patients, the result is thought to be mediated predominately by concurrent CTC AR-V7+ [39].

### 3.2. Activating Mutations in AR

#### 3.2.1. AR Point Mutations

Point mutations in the hinge region or the LBD, which result in reduced ligand specificity and increased trans-activation, are commonly found in mCRPC [67]. CTC DNA studies showed that mutations L702H, T878A, H875Y, W742C, and W743L are the most prevalent mutations with a median of 6 alterations per patient [68]. In the presence of certain mutations, even weaker signals and non-canonical steroid ligands can activate AR [48,69]. H875Y and T878A mutations resulted in activation of the AR pathway promiscuously by estrogens and progesterone while T877A, H875Y, L701H, and L702H mutations resulted in activation by glucocorticoids [69].

Some mutations convert AR antagonists into potent agonists. CTC DNA studies in mCRPC patients resistant to apalutamide or enzalutamide, as well as animal studies, have demonstrated that the F877L mutation converts enzalutamide and apalutamide into agonists [46,47]. F877L mostly co-occurs with the T878A alteration in the endogenous AR allele of the LNCaP cell line upon prolonged exposure to enzalutamide [70]. While enzalutamide is a weak partial agonist of AR-F877L, it becomes a strong partial agonist with double mutant AR-F877L/T878A [49]. However, structurally diverse ARSIs such as abiraterone and galeterone can completely antagonize AR-F877L, as well as the AR-F877L/T878A mutants. F877L, L702H, and T878A mutations mediate abiraterone resistance [71]. In preclinical studies, darolutamide was able to retain antagonistic properties against many clinically relevant AR mutations (F877L, W742L, T7878A) thought to confer resistance to antiandrogen therapies [70]. In addition, darolutamide is a full antagonist to the W741L and T877A mutations, which mediate bicalutamide resistance, and to F876L mutations, which mediate enzalutamide and apalutamide resistance. In an in vitro study assessing response of ARAs to 68 AR mutations in men with CRPC, darolutamide retained efficacy in all gain-of-function AR-FL mutations except A587V [48]. In contrast, enzalutamide caused full or partial activation of 8 mutant types. Nonetheless, it is unclear how this in vitro advantage of darolutamide will translate into clinical context with multiple genetic alterations present in a tumor, poor in vivo bioavailability of darolutamide, and other studies showing cross-resistance to the drug by mechanisms involving AKR1C3/ARV-7 pathway [64]. Of interest, CTC DNA analysis showed that three patients treated with apalutamide acquired F876L mutation on CTC DNA analysis, that was absent prior to treatment. All of these patients had an elevated PSA, but nearly 50% of patients in the entire study population had ≥50% PSA reduction [24]. The clinical relevance of the antagonist-agonist switch mechanism has itself been questioned. If point mutations convert ARSI into potent agonists, withdrawal of the agent should result in improvement in PSA and clinical status. In a study of 47 patients whose disease progressed after enzalutamide treatment, only 5 experienced anti-androgen withdrawal syndrome which was of short duration [72]. Hence, the co-existence of other alterations could impact the outcomes.

TRC253 is a high-affinity competitive binder of wild-type (WT) and mutant AR with proven efficacy in F877L mutant mice models. The drug is currently being studied in a phase I trial and is enrolling for the dose-expansion phase including patients with an F877L mutation [50,51].

PROTACs are protein degrading agents, of which, ARV-110 is currently being evaluated in a phase I/II trial in mCRPC. It degrades the AR in PCa cell lines and animal models with high potency leading to lower expression of PSA [52,53]. This drug produces efficient ubiquitination and degradation of AR by the proteasome and consequent apoptosis in AR-dependent cells. AVR-110 also reduced AR-target gene expression in enzalutamide-resistant tumor models, targeted wild type AR (WT-AR), and amplified AR with T878A, H875Y, F877L, and M895V mutations, but not in tumors with L702H or AR-V7 mutations. Interim results supported a further dose escalation with PSA reduction ≥50% in two out of five patients with T877A and H874 mutations and 2 patients with wildtype AR [73]. ARCC-4 is another PROTAC, derived from enzalutamide, which was shown to degrade AR in VCaP cell lines which exhibit resistance mechanisms including AR amplification, AR-V7, F876L, W741L, M896V and T877A mutations [54].

#### 3.2.2. AR Overexpression

Approximately 80% of patients with CRPC have marked increase in AR mRNA and protein expression [74,75,76,77]. Increased AR expression through gene amplification is considered the mechanism responsible for progression with ADT in about 30% of the patients. AR overexpression coexists with AR point mutations in about 18% of the patients [74,75,78,79,80]. Nearly 80% of the tumors with AR overexpression show elevated AR gene copy numbers and 30% have high-level amplifications. AR gene amplification is more common in CRPC compared to CSPC and has a poor impact on PFS and OS [7,81].

Although in most cases (92%), AR amplification in CTC DNA corresponds to amplification in matched solid biopsy samples, exceptions do occur. A 10-fold AR amplification was detected on CTC DNA analysis in a patient with bone and lymph node metastasis while the same was not detected in a corresponding lymph node biopsy. It was postulated that the tumor clones with high AR amplification were localized to sites of bony metastasis [40]. AR amplification is also more common in patients that progressed on enzalutamide compared to abiraterone or other agents (53% vs. 17% or 21%; *p* = 0.02) [41]. AR amplification is currently being evaluated as a predictive biomarker for low 177Lu-PSMA-617 activity [43]. Mechanistically, AR inhibition upregulates PSMA expression which leads to higher uptake of PSMA-ligand drugs such as 177 Lu-PSMA-617 and increased PSMA tracer uptake on PET in patients with PCa. [82,83,84,85,86,87] In contrast, AR amplification downregulates the PSMA-encoded FOLH1 gene expression, which reduces transcription of PSMA and decreasing PSMA expression [88]. Patients with AR gain were 2.4 times less likely to have a PSA response with PSMA-ligand therapy. Eighty percent of patients with AR gain had early disease progression compared to 20% with normal AR copy levels. PFS was inferior in patients with raised AR compared to normal AR (median 4.7 months vs. 9.4 months; *p* = 0.020), and a similar pattern was seen with OS (median 7.4 vs. 19.1 months; *p* = 0.020). Another study with 66 patients who received radioligand therapies (177Lu-PSMA-617, 177Lu-J591 and 225Ac-J591) showed that 47% had AR amplification or resistant mutations. These patients were less likely to experience a PSA decline ≥30% compared to wild type; it was also associated with inferior OS (median 12.4 vs. 21 months; *p* = 0.043) [44].

AR overexpression can also occur without gene amplification by stabilization of the mRNA or protein or by increasing transcription rates [89,90,91]. This increase could be mediated by the AR gene, expression of c-MYC, or other oncogenes [92,93,94]. AR overexpression results in tumor growth despite minimal androgen stimulation [79,95]. In vitro models demonstrated conversion of bicalutamide to an AR agonist in presence of this aberration [42]. On the other hand, episodic exposure to supraphysiologic doses of testosterone can produce downregulation of AR and potential resensitization to ADT.

The phase II TRANSFORMER trial compared the efficacy of bipolar androgen therapy (BAT) by cycling polar extremes of near-castrate and supraphysiologic testosterone levels with enzalutamide in asymptomatic men with CRPC after progression on abiraterone. Differences in PFS and OS were not statistically significant with either approach. However, patients who underwent BAT followed by enzalutamide had better PFS2 compared to enzalutamide followed by BAT (median 28.2 vs. 19.6 months, HR 0.44, 95%CI 0.22–0.88). The OS was also superior in the BAT followed by enzalutamide group compared to the enzalutamide alone group (median 37.1 vs. 28.6 months, HR 0.52, 95%CI 0.29–0.96). In this study, 38% of the entire study population overexpressed AR and 9% had AR-V7 in CTCs. These aberrations conferred numerically shorter PFS and OS on BAT and enzalutamide therapies; however, the study was not powered to determine if these aberrations could be used as treatment selection biomarkers [30]. It is possible that BAT can extend the PFS on enzalutamide after progression with abiraterone; however, randomized controlled trials are required to confirm this hypothesis. Another approach to eliminate selective pressure on AR from continuous use of one ARSI is by adaptive therapy where patients are switched between on- and off-cycles of treatment based on the PSA and tumor volume. An interim analysis of adaptive abiraterone therapy among 15 patients with mCRPC demonstrated a median rPFS of at least 30 months using only 49% of the conventional continuous abiraterone therapy dose [45].

Bromodomain and extra-terminal chromatin readers (BET) inhibitors including miverbresib and ZEN-3694 regulate AR gene transcription to decrease AR expression and AR-V7 signaling, which results in tumor suppression [96]. Single agent use of miverbresib in a phase I trial did not show a trend that was consistent with a clinical response; and hence, trials with combination of BET inhibitor and enzalutamide are ongoing [34,35]. Phase Ib/IIa results of combination ZEN-3694 plus enzalutamide in 75 patients who progressed on abiraterone (40%), enzalutamide (45.3%), or both (11%) showed a median composite radiographic and clinical PFS of 5.5 months with a 3.5-month mean duration of treatment on the combination [36].

### 3.3. AR Crosstalk with OTHER Signal Transduction Pathways

Resistance to AR-targeted agents in PCa can also be mediated by activation of alternate signaling pathways that are induced by peptide growth factors, PI3K/AKT/mTOR pathway, glucocorticoid receptor (GR) pathway, and through restoration of downstream signaling by alternative proteins.

DNA repair pathway: Patients with tumor cells harboring pathogenic or likely pathogenic variants of BRCA1 or BRCA2 benefit from PARP inhibitors such as olaparib or rucaparib. Although data is limited, these agents can also be used with other homologous recombinant repair (HRR) gene defects such as ATM, BARD1, BRIP1, CDK12, CHEK1, CHEK2, FANCL, PALB2, RAD51B, RAD51C, RAD51D, or RAD54L [97]. PARP-mediated repair pathways are upregulated upon AR inhibition by bicalutamide and enzalutamide and act as a mechanism for PCa cell survival. This process occurs due to ARA-induced unresolved DNA damage, and the pathway could be effectively downregulated by the addition of PARP-inhibitors [98]. In addition to sensitizing PCa cells to DNA damage, PARP-inhibitors also sensitize them to androgen depletion [99]. Olaparib was combined with abiraterone in a phase II trial among 142 patients with mCRPC after progression on prior docetaxel. Approximately 15% of the patients had CTC DNA HRR gene defects. The combination treatment significantly improved rPFS (median 13.8 vs. 8.2 months, HR 0.65, 95%CI 0.44–0.97). As expected, grade 3 and 4 adverse events were common among the combination therapy group (54% vs. 28%) [100]. A phase II trial evaluating the efficacy of abiraterone with veliparib vs. abiraterone with placebo irrespective of HRR gene defect status showed no significant difference in PSA response (72% vs. 64%) or PFS (median 11 vs. 10.1 months). Of note, the patients whose tumors harbored a defective HRR gene (27%) had higher radiology response rates (88% vs. 38%) and PSA response rates (90% vs. 56%) compared to those without HRR defects [101]. Other ongoing trials evaluating the potential benefits of combined therapy include PROPEL (olaparib with abiraterone), MAGNITUDE (niraparib with abiraterone), and TALAPRO-2 trials (talazoparib with enzalutamide) [102,103,104].

PI3K/AKT/mTOR pathway: The PI3K/AKT/mTOR pathway is commonly altered in PCa and signaling can be activated by enzalutamide through stabilization of AKT phosphatase [105,106,107]. Loss of function or deletion of the tumor suppressor was found in approximately 60% of the CRPC patients while mutations that activate PIK3CA mutations occurred in around 30% [108,109]. In preclinical models with PTEN loss, AR and PI3K/AKT pathways maintained tumor survival by reciprocal feedback leading to enzalutamide and abiraterone resistance. Dual inhibition of both the pathways is more effective than blocking either of them [108,109]. A phase III trial with 1101 patients used an AKT inhibitor, iptasertib. Those who were randomized to ipatesertib-abiraterone or ipatesertib-placebo showed that median rPFS was significantly improved in patients with tumors showing PTEN-loss by immunohistochemistry (*n* = 521, median 19.1 vs. 14.2 months, HR 0.65, 95%CI 0.45–0.95) or PIK3CA/AKT1/PTEN-alterations by next generation sequencing (*n* = 205, median 19.3 vs. 14.1 months, HR 0.63, 95%CI 0.44–0.88) [110]. Another AKT-inhibitor, capivasertib, combined with enzalutamide in patients who previously received ARSI showed a positive response in 3 of the 15 patients. All three had PTEN loss or activating AKT mutations [111,112]. Other chemotherapies have been investigated in mCRPC setting: PI3K inhibitor sonolisib (PX-866), AKT/mTOR inhibitor GSK2141795 and combination therapies with PX-866 plus abiraterone, and PI3K inhibitor BKM120 plus enzalutamide. However, none of these studies resulted in meaningful clinical outcomes [113,114,115]. In these combination trials, as well as with the use of the mTOR inhibitor everolimus and the dual mTOR inhibitor MLN0128, the rapid rise in PSA was reversed when treatment was discontinued, which, further confirms a crosstalk between multiple signaling pathways [6,116,117]. Samotolisib, a dual PI3K/mTOR inhibitor plus enzalutamide in a phase Ib/II trial among men with mCRPC showed statistically significant improvement in median serological and radiographic PFS compared to enzalutamide alone (median 2.9 vs. 3.7 months, HR 0.66, 95%CI 0.43–0.99) [118]. GSK2636771, a selective PI3Kβ inhibitor in a phase I/II trial, demonstrated a durable response in 3 of the 12 mCRPC patients, all, of whom had tumors harboring PIK3CB mutations [119].

BRAF-MAPK pathway: Alternative signaling through the MAPK pathway was identified as a potential growth pathway in 2 patients with enzalutamide resistant PCa harboring a BRAF-K601E mutation [120,121]. Pharmacologic inhibition of BRAF or downstream components of MAPK pathway along with AR inhibition resulted in significant inhibition of cell proliferation. With 90% of metastatic PCa harboring alterations in MAPK pathway, larger studies may help us understand if this synergism is reproducible in the clinical setting [122]. BRAF mutations, on the other hand, occur in only about 2% of PCa patients. CXCR7, an atypical chemokine receptor, is one of the most upregulated genes in enzalutamide resistant PCa cells. CXCR7 is repressed by AR but expression increased upon ADT initiation, leading to activation of MAPK/ERK signaling [123]. MAPK inhibitors were able to block CXCR7 downstream pathways, however, resistance developed rapidly. Similarly, increased ERK1/2 expression was seen in tissue samples of men with CRPC. ERK is the immediate downstream target of MEK1/2, and trametinib, a MEK inhibitor, elicited biochemical and clinical responses in a heavily pretreated mCRPC patient [124]. AR-tropomyosin receptor kinase (TRK) crosstalk mediated through nerve growth factor (NGF) also promoted tumor growth in ARSI challenged PCa cell lines, and are targetable by NTRK1/TRKA inhibitors [125]. However, the clinical utility of these inhibitors is limited as NTRK mutations are rarely detected in prostate cancer [126].

Wnt signaling pathway: PCa cells can also gain the ability to synthesize and secrete specific ligands and receptors that help sustain survival through the wnt-beta-catenin pathway independent of androgen signaling [127]. A study among 137 mCRPC patients who developed resistance to enzalutamide or abiraterone found that 11% developed activating mutations in CTNNB1, APC, or RNF43, which are involved in wnt-beta catenin pathway, and that these mutations conferred shorter OS. Interestingly, the CTNNB1 mutations were found only in enzalutamide treated patients. Beta-catenin signaling causes downstream upregulation of hypoxia inducible factor-1 alpha (HIF1α) and vascular endothelial growth factor (VEGF), which promotes angiogenesis. However, targeting mCRPC by tyrosine kinase inhibitors as a monotherapy has not resulted in any clinically beneficial outcomes [111,112]. Though, the HIF1α inhibitor NLG207 in combination with enzalutamide and CCS1477 (inhibitor of the HIFα-AR coactivator CBP-p300) is being evaluated for suppressing the AR-HIFα pathway in patients previously treated with enzalutamide [128,129,130].

Glucocorticoid receptor (GR) activation: Enzalutamide resistance has been attributed in some cases to increased GR expression, which can drive transcription of AR-related genes [131,132]. GR expression was found in only 30% of CSPC but, expression increased after ADT [133]. Of note, AR and GR share the same chromatin binding sites and GR can regulate genes in the AR-pathway [134]. Interestingly, mechanistic studies showed that resistance to enzalutamide can be mediated by increased GR (or other nuclear steroid receptor expression) after exposure to the drug [131]. By overcoming the ligand deficiency conferred by ADT, and regulating AR target genes, the GR-bypass model is a potential resistance mechanism. Loss of TLE3, a transcriptional corepressor, leads to increased GR expression and is implicated in apalutamide and enzalutamide resistance [131]. However, the phase I/II open-label trial of enzalutamide combined with mifepristone, a GR antagonist, in patients with mCRPC showed no benefit in delaying time to PSA-P compared to enzalutamide alone (HR = 1.34, *p* = 0.395) [135,136]. Another phase I/II trial that assessed a selective GR antagonist, CORT125281, plus enzalutamide in mCRPC patients whose cancer progressed on abiraterone is ongoing [137]. In patients who developed progressive disease on abiraterone-prednisone, switching the steroid from prednisone to dexamethasone resulted in PSA decline ≥50% in 34.6% of patients with a median rPFS of 11.8 months [138]. The lower equivalent GR and mineralocorticoid receptor activity of dexamethasone compared to prednisone is postulated as a possible mechanism. Patients with AR gain detected in plasma CTC, however, did not respond to the switch.

Neuroendocrine differentiation: Treatment-related neuroendocrine differentiation is quite prevalent in mCRPC. Studies showed that 16.9% of patients who had disease progression after ADT demonstrated small cell histology on biopsies of metastases [139]. Development of small cell PCa confers a poor prognosis with an OS of 36.6 months compared to 44.5 months in patients with adenocarcinoma (HR 2.02, 95%CI 1.07–3.82). TP53 and RB1 loss can occur as adaptative mechanisms to selective pressures on AR and result in AR independence in the tissue. Overexpression of n-myc and cell-cycle kinase Aurora kinase-A, which drive AR-independent progression by lineage plasticity, was identified in metastatic neuroendocrine PCa [140]. A phase II trial of Alisertib, an inhibitor of ARORA kinase-A and n-myc, in neuroendocrine PCa included 34% of patients who progressed on prior ARSI therapy. Although exceptional responders were identified among patients with genomic amplification of MYCN and AURKA; overall, the study did not meet the primary end-points of 6-month PFS (13.4%) or OS benefit (9.5 months). Inhibition of epigenetic modifiers such as EZH2 were evaluated for their ability to potentially restore sensitivity to ARSIs. Tazemetostat, an EZH2 inhibitor currently used in epithelioid sarcoma and follicular lymphoma, and CPI-1205 are being evaluated in phase I/II trials [23,141]. A phase I trial of a combination of CPI-1205 plus cobicistat (a CYP3A4 blocker) with enzalutamide (after progression on abiraterone) or abiraterone (after progression on enzalutamide) Showed a PSA decline of ≥80% in 14.7% of patients. This PSA decline was mainly confined to the AR-V7 negative group. The phase II part of the trial comparing CPI-1205 with or without enzalutamide has begun.

PD-1/PDL-1 immunoinhibitory pathway: Higher expression of PDL-1 in patients with enzalutamide-resistant PCa prompted the phase III IMbassador250 trial that combined enzalutamide with the PDL-1 blocker atezolizumab in mCRPC after progression on abiraterone or chemotherapy [142]. However, the trial was discontinued due to high toxicity and no improvement in OS [143]. Another trial evaluating a combination of enzalutamide with pembrolizumab in untreated mCRPC patients is ongoing [144].

## 4. Conclusions

Significant advantages have been made in the management of CRPC with second-generation androgen receptor antagonists and androgen synthesis inhibitors. However, the benefits are often short-lived due to the rapid development of resistance to these drugs. Extensive studies on the resistance mechanisms have opened the way to new drug developments which are aimed at reducing the emergence of resistant clones as well as targeting them. These drugs are still in the pipeline with clinical utility being evaluated in numerous clinical trials. Although preclinical data have been promising, many agents were not clinically beneficial. This result is possible because of the interaction of multiple crosstalk pathways and genetic aberrations occurring concurrently, which makes targeted monotherapies less effective. Further understanding of the nuances of resistance mechanisms and wider utilization of clinical trials can help in development of these agents.

## Figures and Tables

**Table 1:** Clinical trials that led to U.S. Food and Drug Administration approval of androgen receptor signaling inhibitors.

Trial Name	Phase	Study Arm	Comparator Arm	Total Patients	Patient Population	Median OS (Months)	HR (95%CI or *p*-Value)	Reference
COU-AA-301	III	Abiraterone + ADT	Placebo + ADT	1195	mCRPC post-docetaxel	15.8 vs. 10.9	0.65 (0.54–0.77)	[9]
COU-AA-302	III	Abiraterone + ADT	Placebo + ADT	1088	Chemo-naïve mCRPC	34.7 vs. 30.3	0.81 (0.70–0.93)	[10]
LATITUDE	III	Abiraterone + ADT	Placebo + ADT	1199	De novo mCSPC	53.3 vs. 36.5	0.66 (0.51–0.76)	[11]
AFFIRM	III	Enzalutamide + ADT	Placebo + ADT	1199	mCRPC post-docetaxel	18.4 vs. 13.6	0.63 (0.53–0.75)	[12]
PREVAIL	III	Enzalutamide + ADT	Placebo + ADT	1717	Chemo-naïve mCRPC	32.4 vs. 30.2	0.71 (0.60–0.84)	[13]
PROSPER	III	Enzalutamide + ADT	Placebo + ADT	933	nmCRPC	67 vs. 56.3	0.73 (0.61–0.89)	[14]
ENZAMET	III	Enzalutamide + ADT	Placebo + ADT	1125	Denovo mCSPC	62% vs. 34% (3-year survival)	0.67 (0.52–0.86)	[15]
SPARTAN	III	Apalutamide + ADT	Placebo + ADT	1207	nMCRPC	73.9 vs. 59.9	0.78 (0.016)	[16]
TITAN	III	Apalutamide + ADT	Placebo + ADT	1052	Denovo mCSPC	NR vs. 52.2	0.65 (0.53–0.79)	[17]
ARAMIS	III	Darolutamide + ADT	Placebo + ADT	1509	nmCRPC	83% vs. 77% (3-year survival)	0.69 (0.53–0.88)	[18]

Abbreviations: CSPC—castration sensitive prostate cancer, mCRPC—metastatic castration resistant prostate cancer, nmCRPC—non-metastatic castration resistant prostate cancer.

**Table 2:** AR-mediated androgen receptor signaling inhibitor resistance mechanisms and drugs with potential action.

Aberration	Mechanism	Treatments Potentially Resistant	Drugs with Potential Action and Active Clinical Trials
AR variants	Lack of ligand binding domain		
AR-V7		Abiraterone, Enzalutamide, Apalutamide, Darolutamide * [28,29] Bipolar androgen therapy [30]	Taxane chemotherapy * [31] TAS3681 [32] EPI-7386 [33] Miverbresib + enzalutamide [34,35] ZEN-3694 + enzalutamide [36]
AR-V567es		Enzalutamide [37,38]	Taxane chemotherapy [39]
AR overexpression	Gene amplification, stabilization of mRNA/protein, increasing transcription rates	Enzalutamide, Abiraterone [40,41,42] 177Lu-PSMA-617, 177Lu-J591, 225Ac-J591 [43,44]	Bipolar androgen therapy pretreatment [30] Adaptive abiraterone therapy [45] Miverbresib + enzalutamide [34,35] ZEN-3694 + enzalutamide [36]
AR point mutations	Low ligand specificity, activation by weaker signals and non-canonical steroid ligands, conversion of ARA into agonists.	Enzalutamide, Apalutamide (A587V, F876L, F877L, G684A, K631T, L595M, Q920R, R630Q, T576A, T878A) [46,47] Darolutamide (A587V) [48]	Darolutamide (F876L, F877L, W742L, T787A, W741L, T878A, L702H, H875Y) [48] Galaterone (F877L, T878A) [49] TRC253 (F877L) [50,51]. ARV-110 [52,53] ARCC-4 [54]

* Clinical benefit/resistance proven in clinical trials.
